# Duty-Cycle-Based Pre-Emption Protocol for Emergency Networks

**DOI:** 10.3390/s22010030

**Published:** 2021-12-22

**Authors:** Gayoung Kim, Minjoong Rim

**Affiliations:** 1 Department of Faculty of General Education, Kangnam University, Yongin-si 16979, Korea; dolga2000@kangnam.ac.kr; 2 Department of Information and Communication Engineering, Dongguk University, Seoul 04620, Korea

**Keywords:** duty-cycle MAC protocol, pre-emption, high priority data, preamble, IoT

## Abstract

This paper proposes a new duty-cycle-based protocol for transmitting emergent data with high priority and low latency in a sensor network environment. To reduce power consumption, the duty cycle protocol is divided into a listen section and a sleep section, and data can only be received when the receiving node is in the listen section. In this paper, high-priority transmission preempts low-priority transmission by distinguishing between high-priority preamble and low-priority preamble. However, even when a high priority transmission preempts a low priority transmission such that the high priority transmission is received first, if the sleep period is very long, the delay may be large. To solve this problem, the high priority short preamble and high priority data reduce receiver sensitivity and increase coverage through repeated transmission. If there are several receiving nodes within a wide coverage, the receiving node that wakes up first can receive the transmission, thus reducing the delay. The delay can also be further reduced by alternately reducing the sleep cycle of one node among the receiving nodes that can receive it. This paper shows that emergent data can be transmitted effectively and reliably by reducing the delay of high-priority data to a minimum through the use of preemption, coverage extension, and an asymmetric sleep cycle.

## 1. Introduction

With the recent growth of the IoT environment [1], there are numerous devices that require effective communication protocols for collecting and transmitting various types of data without unnecessary delay or energy consumption. In this context, the MAC protocol needs to transmit data according to the purpose of the application, and the data is prioritized according to the event type. It is necessary to develop an effective protocol that can reduce energy waste and delay due to retransmission by reducing collisions and contention depending on the type of traffic while considering different priority levels [2].

Data that is simply collected or monitored [3] corresponds to a low level of priority [4], and low energy use in the transmission of such data is essential, because it is important that the data can be communicated for a long period of time. For example, in the collection of temperature and humidity, which is a data type with a low level of priority, the most important thing is for the information to be collected for a long time while using little energy. Meanwhile, when data has a high level of priority, it is necessary to inform people about important information obtained in the monitoring and data collection process. In emergency situations, such as a forest fire or an intruder warning, it is more important to transmit data with as little delay as possible than it is to conserve energy.

The data transmission time and energy consumption differ depending on the purpose and priority level of the event in question. Since data with a higher priority level preferentially occupies a channel over other data, the data transmission delay can be minimized.

By preempting the low priority preamble by broadcasting that there is high priority data using the high priority preamble, other nodes with low-priority data are prevented from occupying the channel, thus reducing the delay of data transmission at the high priority level.

The high priority preamble lowers the reception sensitivity and increases the coverage through repeated transmission so that data can be received by multiple receiving nodes such that the first receiving node to wake up can receive the transmission, thereby reducing the delay.

This paper is a protocol that can reduce data delay time according to the difference in importance in various data transmission, which is a characteristic in the IoT environment. By giving high priority to data with high importance, it shows much lower latency compared to other data. Our proposed pre-emption MAC has proven to be an effective mac that can minimize the latency of important data compared to the existing asynchronous MAC.

## 2. Related Works

In the MAC protocol, various methods have been developed to reduce delay. This chapter describes existing MAC protocols [5] that are used to minimize delay.

### 2.1. Access Priority

The goal is to transmit high-priority data without delay according to the importance of the data. To this end, for high-priority data, there is a method that can be used to increase channel access by reducing IFS [6] and CW [7]. There is also a protocol that has a fast data transmission opportunity by adjusting the contention access phase (CAP) [8] according to the priority. The representative protocols are PR-MAC [9] and CoR-MAC [10].

### 2.2. Transmit Opportunity (TXOP)

802.11e MAC [11] has a TXOP [12] that allows any node to transmit data for a certain period of time. Using the concept of TXOP, a certain time can be assigned, or the transmission time can be forcibly suggested.

For the transmission of Quality of Service (QoS) data frames including priority by the contention-based channel access method, Enhanced Distributed Channel Access (EDCA) [13] defines four Access Categories (ACs) [14]. As presented in Table 1, the traffic arriving from the MAC layer with different user priorities in the application program is expressed as the corresponding AC, and differentiated transmission is provided for each AC.

Data transmission contention has a higher priority with smaller values of Arbitration Inter Frame Space (AIFS) and *CW_min_*, respectively, rather than the values of Distributed IFS (DIFS) and *CW_max_* used by the Distributed Coordination Function (DCF). Through this, the channel access delay is shortened and a wider band can be used in the traffic environment.

Frames from the AC with the highest priority are sent first, and the other ACs update the backoff counter again by increasing the contention window value. The initiation of a TXOP occurs when accessing a channel according to EDCA rules.

When two or more frames are piled up in one AC, if EDCA TXOP is obtained, EDCA MAC may attempt to transmit multiple frames. If the QoS station has already transmitted one frame and is able to receive the transmission of the next frame in the same AC as well as an ACK for it within the remaining TXOP time, the QoS station tries to transmit the frame after the Short IFS (SIFS) time interval. The TXOP limit value is passed from the QoS access point to the QoS station. If the size of the data frame to be transmitted exceeds the TXOP limit value, then the QoS station fragments the frame into several small frames and transmits the frame within a range that does not exceed the TXOP limit value.

### 2.3. Resource Reservation

Collision-free data transmission can be achieved by using a slot reservation mechanism to transmit data without delay. DW-MAC [15] and SR-MAC [16] are representative MACs that use a scheduling mechanism to reserve several time slots during a sleep period in which a node can transmit multiple packets.

Demand Wakeup MAC (DW-MAC) is a synchronous MAC protocol in which one cycle is divided into three sections: Sync, Data, and Sleep period. A one-to-one mapping function utilizing the Scheduling Control Frame (SCH) is used for data transmission during the sleep period.

For several transmitting nodes to transmit data packets to one receiving node, they first transmit an SCH frame in the listen start period. Accordingly, the transmitting node can avoid collisions with other transmitting nodes by transmitting data in reserved slots of the sleep cycle according to the pre-reserved scheduling.

### 2.4. Ethernet of Frame Preemption Mechanism

**Figure 1:**
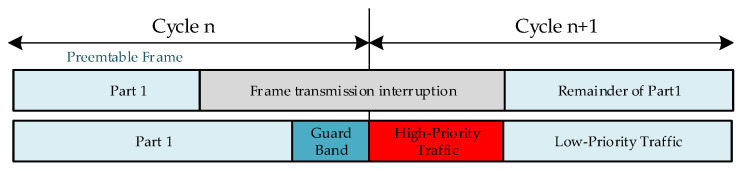
Frame preemption mechanism [17].

As shown in Figure 1, to support frame preemption [17] in the Ethernet environment, two types of traffic are distinguished. The frame type is identified by examining the VLAN tag defined by IEEE 802.1Q. Frames arriving at the MAC client are serviced as either preemptable MAC (pMAC) or express MAC (eMAC), and if frames of both types arrive simultaneously, then the express traffic with higher priority is serviced first.

When an express frame arrives while a preemptable frame is already being transmitted, if a specific condition is satisfied, the current transmission of the preemptable frame is stopped, and the express traffic service is transmitted. Then, the transmission of the interrupted frame is resumed and reassembled by the MAC Merge Sublayer (MMS), which is part of the modified Ethernet MAC that supports the remaining untransmitted preemptable frames.

However, pMAC and eMAC have a problem in that if many frames with different priorities actually arrive at the same time, a collision and data delay occurs.

## 3. System Model

Our proposed Pre-emption MAC supports the successful transmission of data with minimal delay by giving high priority to data that requires urgent transmission depending on the situation involving a massive number of various sensor devices.

**Figure 2:**
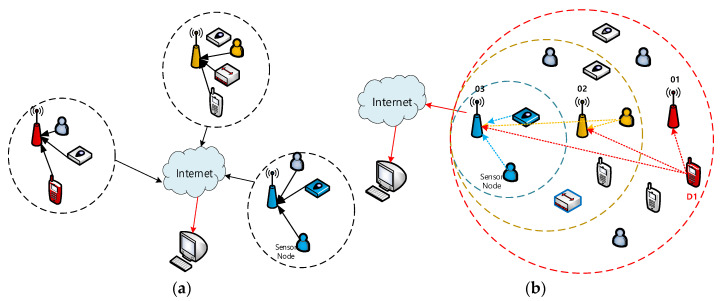
Networks for transmitting various types of data: (**a**) Network without priority data; (**b**) Network with priority data.

Figure 2 shows the data transmission of nodes in a network without priority data and in a network environment with priority data in a massive environment. In Figure 2a below, in the existing sensor network, about *N* various nodes each attempt to transmit data to one coordinator, and each node has its own transmission coverage.

On the other hand, in Figure 2b, *D*_1_ device, which will transmit high-priority data due to the pre-emption protocol, attempts to transmit data before other devices in an environment with priority data. Although other devices can transmit data within their certain coverage, the *D*_1_ device can transmit data directly to a coordinator that can receive data among coordinators 01, 02, and 03.

When there are multiple receiving nodes within a wide coverage, the transmitting node with high priority data transmits to the first awake receiving node, thus reducing the delay.

This section may be divided by subheadings. It should provide a concise and precise description of the experimental results, their interpretation, as well as the experimental conclusions that can be drawn.

**Figure 3:**
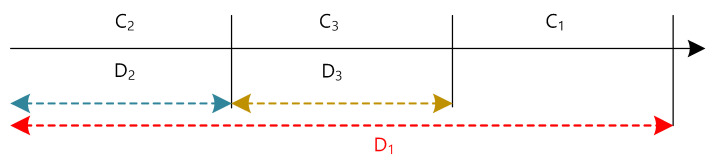
Coverage according to device type.

Figure 3 shows the coverage (*C*_1_, *C*_2_, *C*_3_) according to each respective device (*D*_1_, *D*_2_, *D*_3_). *D*_2_ and *D*_3_ transmit data to the receiving node within the coverage of *C*_2_ and *C*_3_, respectively, but *D*_1_ having relatively high priority data can transmit data up to *C*_1_, *C*_2_, and *C*_3_ in a wide range.

When *D*_1_ transmits *D*_2_ and *D*_3_ having low priority data, the *D*_2_ and *D*_3_ nodes stop transmitting their preamble, and the *D*_1_ data is transmitted preferentially. The high priority preamble of *D*_1_ is also transmitted to the receiving nodes in the ranges of *C*_1_, *C*_2_, and *C*_3_ by repeating transmission. As a result, when there are several receiving nodes within a wide coverage area, the data transmission delay can be reduced by initiating transmission to the receiving node that wakes up the earliest.

## 4. Duty-Cycle Pre-Emption MAC Protocol

### 4.1. Operation of Duty-Cycle Pre-Emption Protocol

**Figure 4:**
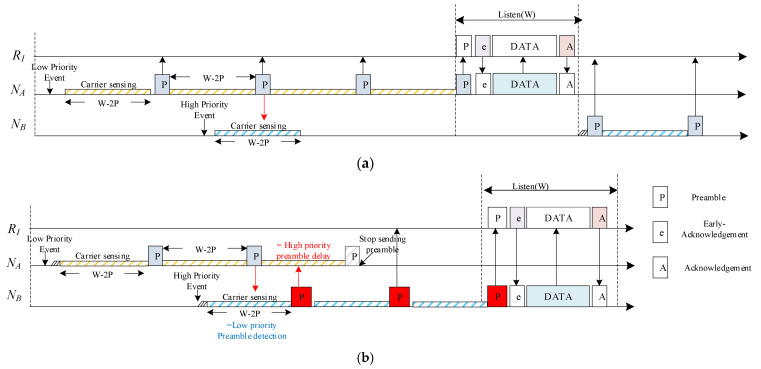
Duty-cycle-based protocol operation methods: (**a**) X-MAC Protocol; (**b**) Pre-emption MAC Protocol.

The operation method of the proposed protocol is illustrated in Figure 4 below. Figure 4a shows the operation method of the asynchronous MAC protocol by which transmitting nodes transmit data. A case in which data with the same priority is transmitted is expressed as a data transmission method similar to a general asynchronous method.

The period in which the carrier sensing is performed is the time obtained by subtracting (*P*) *2—which is the preamble period—from the listen period (*W*). Let us say that the sending node *N_A_* is about to transmit data by generating an event. Carrier sensing is performed during *W*-2*P*, and the preamble is transmitted to the receiving node *R*_1_, followed by carrier sensing again. At this time, the transmitting node *N_B_* attempts to transmit data by generating an event whose priority is higher than that of *N_A_*.

However, since the *N_A_* preamble was detected during the *N_B_*’s carrier sensing period, the carrier sensing is performed again after a certain period of time has elapsed. After all, even if the node has data with high priority, data transmission will be impossible if another node is already occupying the channel when carrier sensing is performed.

Figure 4b shows the data transmission operation method when preamble is applied. The transmitting node *N_A_* performs carrier sensing during *W*-2*P* to transmit data by generating an event having a low priority, and it transmits a preamble to the receiving node *R*_1_ once and performs carrier sensing again. The preamble is continuously transmitted until receiving Early Acknowledgment (Early ACK), which is the response signal of the preamble of the receiving node *R*_1_.

However, when an event having a high priority occurs in the *N_B_* and carrier sensing is performed to transmit data, if the *N_B_* receives the *N_A_* preamble during the carrier sensing period, it can be determined that the node having a lower priority than itself transmits the preamble. The *N_B_* broadcasts the preamble immediately after sensing its own carrier to prevent transmission of the preamble of the *N_A_* having a lower priority than itself, and it thereby acquires the channel.

Through this process, the high-priority *N_B_* node steals the channel from the *N_A_* and starts transmitting its data to the receiving node *R*_1_.

### 4.2. Algorithm of Duty-Cycle Pre-Emption Protocol

**Figure 5:**
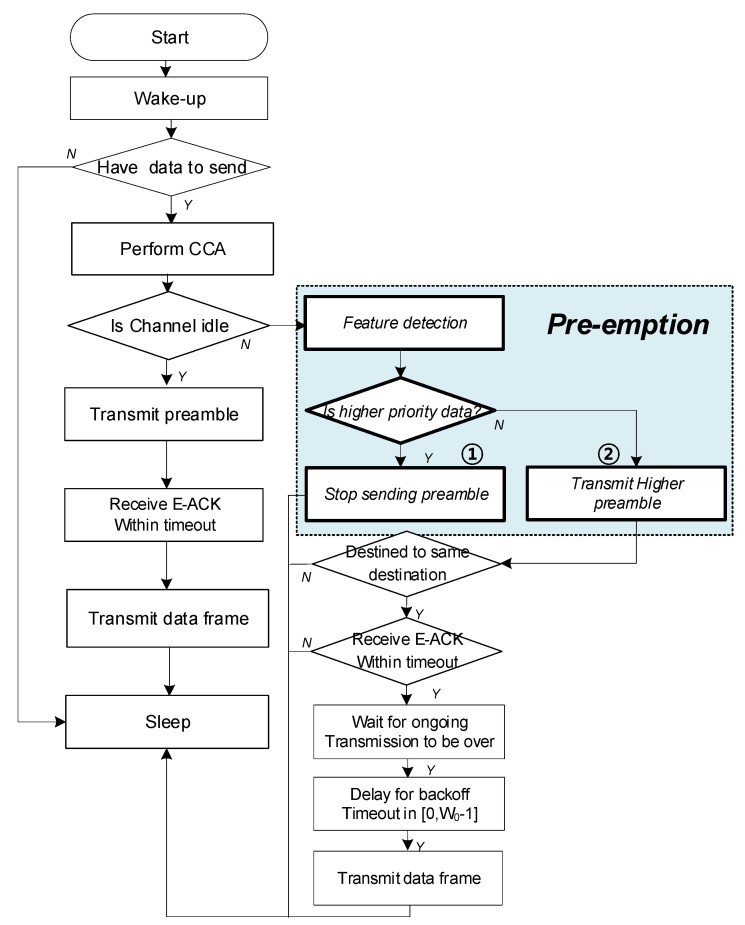
Pre-emption protocol algorithm.

Figure 5 shows the algorithm of our proposed preamble protocol. If a node wakes up and there is data to send, channel sensing is performed for a certain period of time. Meanwhile, if the channel is empty, the preamplifier operates in the blue box.

From the sender’s point of view, if the channel is busy and the result of sensing the channel has a higher priority than the data it has, ① it stops the preamble it is sending and sleeps for a set amount of time.

However, if the data of the node currently transmitting the preamble is the same as the priority of other data, ② the current preamble is transmitted to transmit the data to be sent. If the destinations are the same, a backoff timeout is performed for at least some predetermined time (time slot * contention window size) to avoid collision between preambles, and the data is transmitted.

If there is data to be transmitted continuously between data with high priority, other data cannot be transmitted. Moreover, if there are multiple devices with the same high-priority data to be transmitted, and if data transmission is required at the same time, the first node to preempt the channel through CW transmits. High priority data needs to be processed urgently compared to low priority data, but since it is assumed that the overall data amount is much smaller, there are relatively few nodes with high priority. All nodes work on carrier sensing before starting preamble transmission and between periodic preamble transmission.

Carrier sensing is performed before preamble transmission and if a node with the same or higher priority is transmitting the preamble, it does not transmit the preamble. Carrier sensing is performed before preamble transmission, and if only the node having a lower priority is transmitting the preamble to itself, the preamble transmission is started.

During preamble transmission, if a node with the same or higher priority is transmitting the preamble by performing carrier sensing, the preamble transmission is stopped. When two nodes that want to transmit data have the same priority, if one node starts transmitting the preamble first, the other node does not transmit the preamble by carrier sensing and waits until the node that transmitted first finishes transmitting the data.

If two nodes having the same priority start transmitting the preamble at the same time, a collision may occur. However, if a random offset is given randomly at every cycle in the preamble transmission interval, a node with a larger offset can stop transmission by carrier sensing, thus solving the collision problem.

### 4.3. Determining the Pre-Emption Delay

We use a mathematical model to calculate the latency with our proposed algorithm. Using this model, we compare the performance of the existing duty cycle MAC protocol and the preamble MAC protocol for transporting traffic with different priorities in the IoT environment. 

Assuming that the period in the general duty cycle MAC is *T*, the average delay time required to successfully transmit data is expressed by Equation (1). The related parameter settings are listed in Table 2.

(1) $$D_{n}:\;CW+\frac{T_{NA}}{2}+t_{data}+t_{ack}$$

If there is data with high priority, the average delay time required to successfully transmit data by applying the priority is expressed as Equation (2). If the event of the high level priority node occurs at time *z*, the transmission of the low level priority preamble that was in the process of being transmitted is stopped, and when the high level priority node transmits the preamble and receives the early ACK from the receiving node, data is transmitted immediately.

(2) $$D_{p}:\;CW+\frac{T_{NA}}{2}+(z-\frac{T_{NA}}{2})+\frac{T_{NB}}{2}+t_{data}+t_{ack}$$

The existing Duty Cycle MAC protocol continues to transmit the preamble until the transmitting node—which first acquired the channel—receives an Early ACK from the receiving node regardless of the importance of the data. At this point, the node transmits the preamble.

*D_n_* in Equation (1) can be described as the time *CW* for channel sensing, *T_NA_*/2, which is the average short preamble transmission time transmitted by nodes having the same priority, and the time obtained by adding data and ack. Equation (2), *D_p_* is expressed as the time obtained by subtracting the low-priority preamble transmission time *T_NA_*/2 from the high-priority event occurrence time, which is the time *CW* and *z* time for channel sensing, plus preamble transmission time of node with high priority, *T_NB_*/2 the duration of data and ACK.

As a result, since the node that seized the channel first has to wait until the data transmission is completed, there is a problem in that the delay time is long in the case of urgent data transmission. To solve this problem, we can compare the shortened delay time with our proposed algorithm using Equations (1) and (2).

## 5. Result of Simulation

To validate our proposed pre-emption MAC, we ran the model under various pre-emption MAC configurations and data arrival rates, and compared the estimates of delay, and with simulation results using matlab R2018A.

In the simulation,

1. the network is fully connected,
2. every 100 s one of a node’s neighbors is randomly
  selected as the destination of the packets that arrive
  in the following 100 s,
3. the simulation time is 1000 s, and
4. all the results (delay) are averaged over 30 runs.

Table 3 lists the set values of parameters defined to compare the delay times of the asynchronous MAC protocol and the Pre-emption MAC protocol.

**Figure 6:**
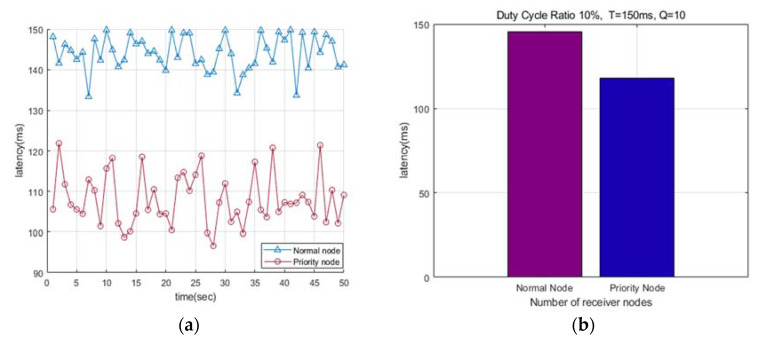
Comparison of latency between Duty Cycle MAC and Pre-emption MAC: (**a**) Comparison of latency over time; (**b**) Comparison of average delay.

Figure 6a presents a diagram comparing the data transmission delay times of asynchronous MAC and our proposed pre-emption MAC. The period of the transmitting and receiving nodes is 150 ms, the duty-cycle ratio (DC) is 10%, and the transmission delay time is shown according to time.

The existing duty cycle MAC does not have a wide data transmission range, and it can only be transmitted within one receiving node. In the pre-emption MAC, the transmission range is wide, so transmission is possible to the first node among multiple receiving nodes whereas it is impossible from other transmitting nodes. Therefore, the latency is low.

According to the experiment, in the case of pre-emption MAC, when there are two receiving nodes, it has less latency than the duty cycle MAC. Figure 6b compares the average delay times of asynchronous MAC and pre-emption MAC, and the pre-emption MAC shows 15% lower latency on average.

**Figure 7:**
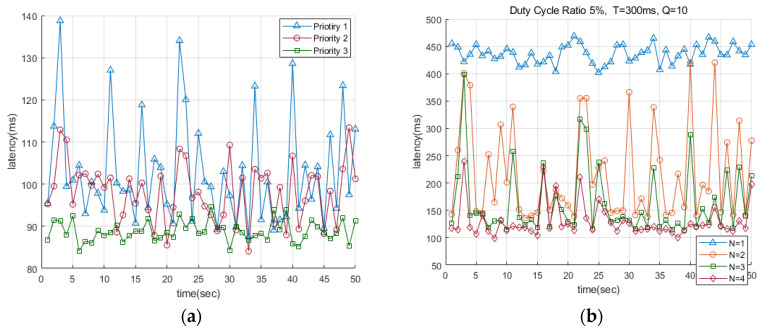
Comparison of latency Pre-emption MAC: (**a**) Delay time of various pre-emption MAC; (**b**) Delay time according to the number of receiving nodes.

Figure 7a shows that the total number of receiving nodes and the total number of wake-ups are the same in the pre-emption MAC protocol, but that the number of wakeups is different for each receiving node; because of this, although the same amount of energy is consumed, there are different delay times for successfully transmitting the data.

Priority 1 has three receiving nodes with 10% DC; Priority 2 has two receiving nodes with 15% DC and one receiving node with 7.5% DC; and Priority 3 has one receiving node with 30% DC. Considering a total time of 600 ms, the receiving nodes wake up 12 times.

Among the three cases, Priority 3 shows the lowest delay time, and the delay time can be reduced more effectively when there is one receiving node than it can be when there is a large number of other receiving nodes. As a result, it was possible to determine a way to achieve low latency by varying the number of receiving nodes and the number of wake ups while consuming the same total amount of energy.

Figure 7b shows a graph expressing the delay time according to the number of receiving nodes when the total period is 300 ms and DC is 5%. Since the normal duty cycle MAC has one receiving node (*N =* 1), the transmission delay time shows a relatively higher delay time than the pre-emption MAC. For example, in the case of the duty cycle MAC with one receiving node (*N =* 1) and the pre-emption MAC with four receiving nodes (*N =* 4), about 5 times faster latency was confirmed on average. In the case of the pre-emption MAC, it was confirmed that the wider the coverage, the more it can transmit to multiple receiving nodes, and the delay can be reduced because the first waking node among the receiving nodes that can receive the data does so.

**Figure 8:**
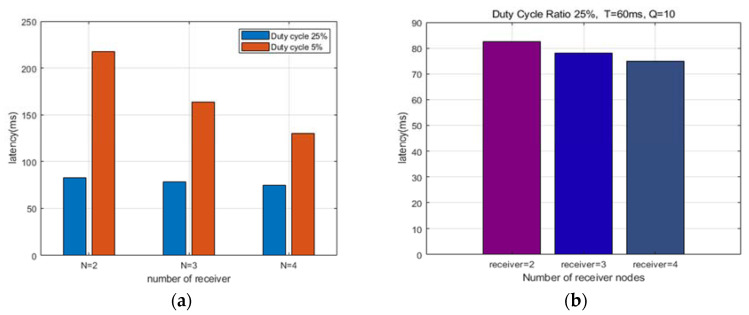
Comparison of latency Pre-emption MAC: (**a**) Comparison of latency according to duty cycle ratio; (**b**) Comparison of average latency according to the number of receiving nodes.

Figure 8a shows a graph comparing the average delay time according to the number of receiving nodes and DC in pre-emption MAC. When the DC is 25%, it is confirmed that there is little change in the delay time even if there is an increase in the number of receiving nodes.

However, when the DC is 5% and the number of nodes is 4, the delay time can be reduced by about 1.67 times compared to when the number of nodes is 2 (*N =* 2) and when the number of nodes is 4 (*N =* 4). When the DC of the receiving node is low, the delay time can be affected substantially as the number of receiving nodes increases, but when the DC is relatively high, the number of receiving nodes is hardly affected.

Figure 8b shows the average delay time according to the change in the number of receiving nodes when the DC of the receiving node is 25%; it is a re-expression of what was described in Figure 8a. The figure confirms that the delay time was improved by about 9% when comparing the case with two nodes and the case with the number of nodes. These results confirmed that as the duty cycle ratio grows larger, the data transmission time is not significantly affected by the number of receiving nodes.

**Figure 9:**
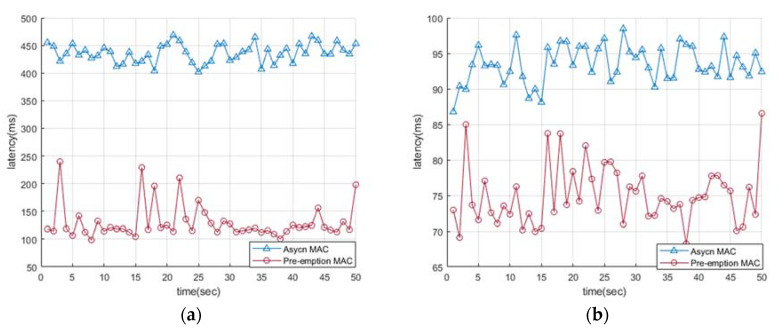
Comparison of latency Async MAC and Pre-emption MAC (**a**) Delay time of duty-cycle ratio (5%); (**b**) Delay time of duty-cycle ratio (25%).

Figure 9a,b shows a diagram comparing the data transmission delay times of legacy asynchronous MAC and pre-emption MAC. The duty cycle ratios in the figures are 5% and 25%, respectively. Both figures present that the transmission delay time is shown according to time.

When the DC is 25%, it is confirmed that there is little change in the delay time even if there is an increase in the number of receiving nodes. Figure 9a,b show that our proposed pre-emption has a shorter delay time than the existing asynchronous MAC although the delay time is different depending on the duty cycle. In conclusion, pre-emption mac can achieve effective results if it is applied to data that requires minimum latency.

## 6. Conclusions and Future Work

This paper shows that, to send emergent data with low latency in the IoT network environment, it is possible to expand the coverage so that it can be transmitted to multiple receiving nodes—thus allowing high priority data to be transmitted before other low priority data—thereby reducing the delay time and improving the performance.

To minimize the delay time of the pre-emption MAC, the optimal environment was presented through various experiments showing that the transmission delay can be reduced in an environment requiring the same energy consumption by changing the number of receiving nodes and changing the duty cycle ratio of the receiving nodes. An attempt was made to reduce the delay time by preempting the low priority transmission when transmitting the high priority preamble through simulation, but there was a problem in that this led to no change in the data transmission delay time, even after the preemption, when the sleep period was very long.

To solve this problem, the receiver sensitivity was lowered and the coverage was increased through repeated transmission of the data transmission of the high priority node. It was possible to reduce the delay time required for data transmission by reducing the waiting time by transmitting to the node that wakes up first among multiple receiving nodes within a wide coverage area. It was also confirmed that the data transmission time was effectively reduced by reducing the sleep period by changing the DC of several receiving nodes.

For example, the simulation results show that, when DC was 5%, the pre-emption MAC was able to transmit data about 5 times faster than the normal duty cycle MAC when there were four receiving nodes. In addition, as the DC ratio is smaller and the number of receiving nodes is increased, the delay time can be effectively reduced.

In future research, we intend to develop an algorithm that can determine the optimal DC and number of receiving nodes that can effectively reduce data latency with minimal energy through deep learning [18,19,20]. We also plan to improve it to the point of being a pre-emption MAC that can be applied in real time in the real-world IoT [21,22,23] environment.

## Figures and Tables

**Table 1:** According to traffic priority AC classification.

Priority	User Priority	802.1D Designation	Access Category (AC)
Low	1	BK	AC_BK
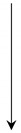	2	-	AC_BK
	0	BE	AC_BE
	3	EE	AC_BE
	4	CL	AC_VI
	5	VI	AC_VI
	6	VO	AC_VO
High	7	NC	AC_VO

**Table 2:** Duty Cycle Pre-emption MAC Protocol Parameters.

Symbol	Quantity
*T*	One period of time of asynchronous MAC
*D_p_*	The section in which the preamble of the node with high priority is transmitted *n* times
*D_n_*	The section in which the preamble of the node with low priority is transmitted *n* times
*CW*	(Contention Window/2) ∗ time slot
*t_eack_*	Early Acknowledgement duration
*t_ack_*	Data Acknowledgement duration
*t_data_*	Data duration
*τ*	Slot time
*T_NA_*	The section in which the preamble of the node with low priority is transmitted *n* times (P∗*n*), ∗ means multiplication
*T_NB_*	The section in which the preamble of the node with high priority is transmitted x times (P∗x)
*P*	Short preamble duration
*n*	Number of cycles
*z*	When the event of the node with high priority occurs

**Table 3:** System Parameters.

Parameter	Value	Parameter	Value
Bandwidth	25 kbps	Duration of E-ACK (*E-ACK*)	3 ms
Transmission Range	100 m	Data Frame Size (*S*)	50 bytes
Carrier Sensing Range	200 m	Transmission Power (*TX*)	52.2 mW
Data Arrival Rate (λ)	1 frame/s	Reception Power (*RX*)	29.1 mW
Duration of Short Preamble (*SP*)	3 ms	Idle Listening Power (*Idle xp*)	52.2 mW
Contention Window Size (*CW*)	16	Sleeping Power (*Sleep xp*)	0.001 mW

## Data Availability

Not applicable.
